# CCL20 induces colorectal cancer neoplastic epithelial cell proliferation, migration, and further CCL20 production through autocrine HGF-c-Met and MSP-MSPR signaling pathways

**DOI:** 10.18632/oncotarget.28131

**Published:** 2021-11-23

**Authors:** Bisweswar Nandi, Jonathan Pastrana Del Valle, Mehmet K. Samur, Allison J. Gibbons, Rao H. Prabhala, Nikhil C. Munshi, Jason S. Gold

**Affiliations:** ^1^Research Service, VA Boston Healthcare System, West Roxbury, MA, USA; ^2^Harvard Medical School, Boston, MA, USA; ^3^Beth Israel Deaconess Medical Center, Boston, MA, USA; ^4^Dana-Farber Cancer Institute, Boston, MA, USA; ^5^Medicine Service, VA Boston Healthcare System, West Roxbury, MA, USA; ^6^Brigham and Women’s Hospital, Boston, MA, USA; ^7^Surgery Service, VA Boston Healthcare System, West Roxbury, MA, USA

**Keywords:** CCL20, CCR6, HGF, MSP, colorectal cancer

## Abstract

CCL20-CCR6 interactions promote colorectal cancer through direct effects on neoplastic epithelial cells and through modulating the tumor microenvironment. The mechanism of these effects on neoplastic epithelial cells is poorly understood. This study demonstrates that CCL20 induces secretion of hepatocyte growth factor (HGF) and phosphorylation of HGF’s cognate receptor c-Met in HT29 and HCT116 colorectal cancer cell lines both in concentration- and time-dependent manners. Similar to CCL20, HGF induces migration, autofeedback CCL20 secretion, and ERK1/2 phosphorylation in the colon cancer cells. CCL20-dependent ERK1/2 phosphorylation is blocked by HGF inhibition, and CCL20-dependent migration and CCL20 secretion are blocked by inhibition of HGF or ERK. Interestingly, unlike CCL20, HGF does not induce proliferation of colon cancer cells, and CCL20-dependent cell proliferation is not blocked by direct HGF inhibition. CCL20-dependent proliferation, however, is blocked by the multi-tyrosine kinase inhibitor crizotinib. Exploring this effect, it was found that CCL20 also induces production of MSP and phosphorylation of MSP’s receptor MSPR by the colorectal cancer cells. CCL20-dependent cell proliferation is inhibited by directly blocking MSP-MSPR interactions. Thus, CCL20-mediated migration and CCL20 secretion are regulated through a pathway involving HGF, c-Met, and ERK, while CCL20-mediated proliferation is instead regulated through MSP and its receptor MSPR.

## INTRODUCTION

The tumor microenvironment contains a rich signaling network of soluble factors including cytokines, chemokines, growth factors, and cellular metabolites. These factors mediate crosstalk between neoplastic epithelial cells and various stromal cells including leukocytes, fibroblasts, stem cells, endothelial cells, and pericytes. In addition to mediating paracrine signaling between cell types, soluble factors in the tumor microenvironment can also act in an autocrine fashion by exerting effects on the same cell type responsible for their production. Soluble factors in the tumor microenvironment are essential for several tumor-promoting processes including tumor cell proliferation; prevention of tumor cell death; migration of cells into, out of, or within the tumor; metastasis; immune escape; and therapeutic resistance.

Chemokines are a family of small signaling proteins with shared structural characteristics. They are involved in the development and progression of many cancer types, including colorectal cancer. Chemokines are most well known for their ability to induce immune cell migration and chemotaxis but can also have also direct effects on non-immune cells including tumor-promoting effects on neoplastic cells. Expression of the C-C chemokine CCL20, also known as macrophage inflammatory protein-3 α (MIP-3 α) and liver activation regulated chemokine (LARC), as well as its receptor CCR6 have been observed in several malignancies including colorectal cancer. CCL20 is the sole known ligand for CCR6, which in turn, is the only known receptor for CCL20. It has been shown that high serum CCL20, tumor CCL20, and tumor CCR6 expression correlate with poor prognosis of colorectal cancer in human patients [1–6]. Several animal studies have also confirmed the importance of these molecules in this disease [1, 2, 7–11].

While the effects of CCL20-CCR6 interactions in colorectal cancer have largely been thought to be mediated by stromal elements such as macrophages [8, 9]; regulatory T cells (Tregs) [7, 11]; B cells and γδ T cells [10]; and blood vessels [2], several studies have also demonstrated the importance of direct effects on neoplastic epithelial cells. CCL20 has been observed to increase proliferation [1, 8, 12–15], increase migration [12, 16, 17], and initiate an auto-feedback loop by inducing further secretion of CCL20 [8] in human colorectal cancer cell lines *in vitro*. Furthermore, in immunosuppressed xenograft mouse models, where many stromal effects should be abrogated, it has been shown that blockade of CCL20-CCR6 interactions decreases colorectal tumor growth [18], and that overexpression of CCR6 in the cancer cells promotes metastases [7]. Furthermore, it has been observed that an antibody targeting CCR6 inhibits the growth of syngeneic mouse colon cancers in a CCR6-deficient mouse model where CCR6 expression is thus confined exclusively to the transplanted tumor [9].

The mechanisms through which CCL20-CCR6 interactions elicit their direct effects on neoplastic epithelial cells in colorectal cancer is poorly understood. The aim of this study is to elucidate these mechanisms.

## RESULTS

### CCL20 stimulation induces HGF production and c-Met phosphorylation by colon cancer neoplastic epithelial cells

**Figure 1:**
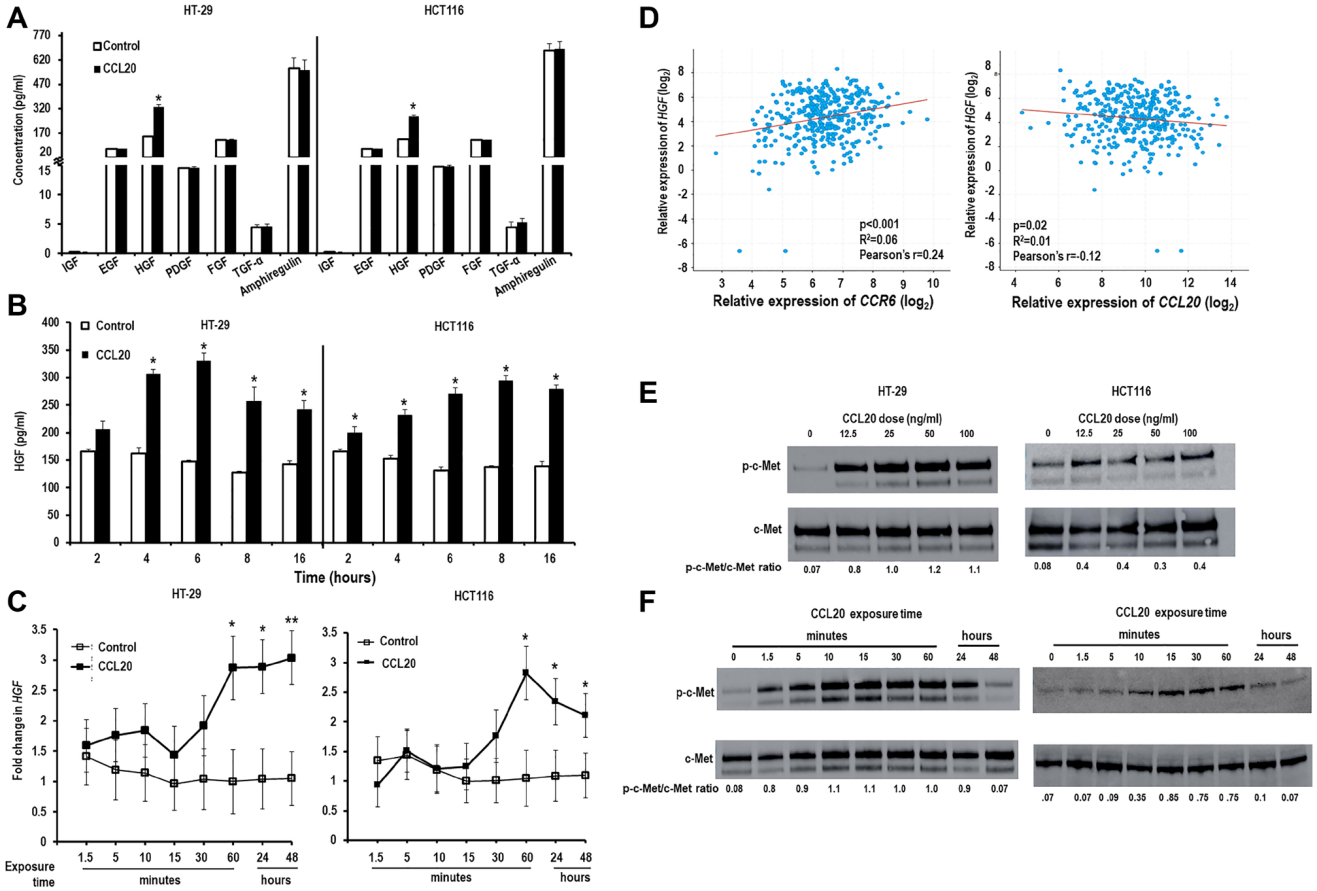
CCL20 induces colorectal cancer cells to produce HGF. Colorectal cancer cell lines HT-29 (left) and HCT116 (right) were stimulated with CCL20 (100 ng/ml), and production of growth factors was assessed by ELISA. For time points after 2 hours, media (including CCL20) was replaced 2 hours before collection times. (**A**) Supernatants were collected at 6 hours. (**B**) Supernatants were collected at times shown. (**C**) Production of HGF by colorectal cancer cells at various time points after stimulation with CCL20 was measured in mRNA of cell lysates by qRT-PCR. (**D**) Correlation between expression of *HGF* and *CCR6* (left) and between *HGF* and *CCL20* (right) in human colorectal cancer was assessed using gene expression data from The Cancer Genome Atlas (TCGA). Activation of the HGF receptor c-MET in colorectal cancer cells after stimulation with CCL20 at various (**E**) concentrations for 10 minutes and (**F**) time points with 100 ng/ml CCL20 was assessed by measuring phosphorylation (phosphorylated c-Met or p-c-Met) using Western blots. ^*^ represents *p* < 0.05 and ^**^ represents *p* < 0.01.

It has been previously demonstrated that CCL20 has direct effects on neoplastic colon cancer cells causing proliferation [1, 8, 12–15], migration [12, 14–17], and initiating an auto-feedback loop by inducing further secretion of CCL20 [8]. The mechanisms through which CCL20 elicits these effects is poorly understood. We first investigated if the effects of CCL20 on colorectal cancer cells are mediated through the secretion of growth factors. For this, the colon cancer cell lines HT-29 and HCT116 were treated with CCL20 and culture supernatants were checked for the secretion of classical cancer cell growth factors such as epidermal growth factor (EGF), fibroblast growth factor (FGF), hepatocyte growth factor (HGF), insulin-like growth factor (IGF), platelet-derived growth factor (PDGF), transforming growth factor alpha (TGF-α), and amphiregulin by ELISA. Only secretion of HGF was found to be significantly increased by both the cell lines in the 4–6 hour time period after stimulation (Figure 1A). TGF-α was also found at a significantly higher level in supernatants from HT-29 cells and amphiregulin in supernatants from HCT116 cells collected from the entire first 48 hours after stimulation with CCL20 (Supplementary Figure 1A). We next explored the kinetics of HGF secretion upon treatment with CCL20. As shown in Figure 1B, HGF production peaked 6 hours after stimulation with CCL20. At the mRNA level, *HGF* transcript expression remained elevated or continued to increase between 1 and 24 hours (2.9 and 2.9 fold for HT-29 and 2.8 and 2.3 fold for HCT116, Figure 1C). In contrast, we did not note upregulation of *TGF-α* or *amphiregulin* after CCL20 stimulation (Supplementary Figure 1B). To further corroborate a link of CCL20 exposure to HGF secretion in colorectal cancer, a correlative analysis was performed between gene expression of *CCL20* and *HGF* as well as between CCL20’s receptor, *CCR6,* and *HGF* and using data from human colorectal cancers collected by the Cancer Genome Atlas (TCGA) (Figure 1D). Interestingly, there was strong positive correlation found between expression of *CCR6* and *HGF* (R^2^ = 0.06, Pearson’s *r* = 0.24). There was a weak negative correlation between *CCL20* and *HGF* (R^2^ = 0.01, Pearson’s *r* = –0.12). These results support a link between CCL20-CCR6 signaling and HGF secretion in human colorectal cancer but suggest that this might be regulated at the level of CCR6 rather than CCL20. (CCL20 and CCR6 expression were also strongly correlated (R^2^ = 0.04, Pearson’s *r* = 0.21, Supplementary Figure 2).) To assess whether CCL20 stimulation of colorectal cancer cells induces activation of HGF’s receptor, c-Met, we measured expression of c-Met and phosphorylated c-Met by Western blots and found CCL20 to induce c-Met phosphorylation after CCL20 exposure in both a concentration-dependent (Figure 1E) and time-dependent manner (Figure 1F) in both HT-29 and HCT116.

### HGF induces colorectal cancer cell migration and CCL20 production but not proliferation

**Figure 2:**
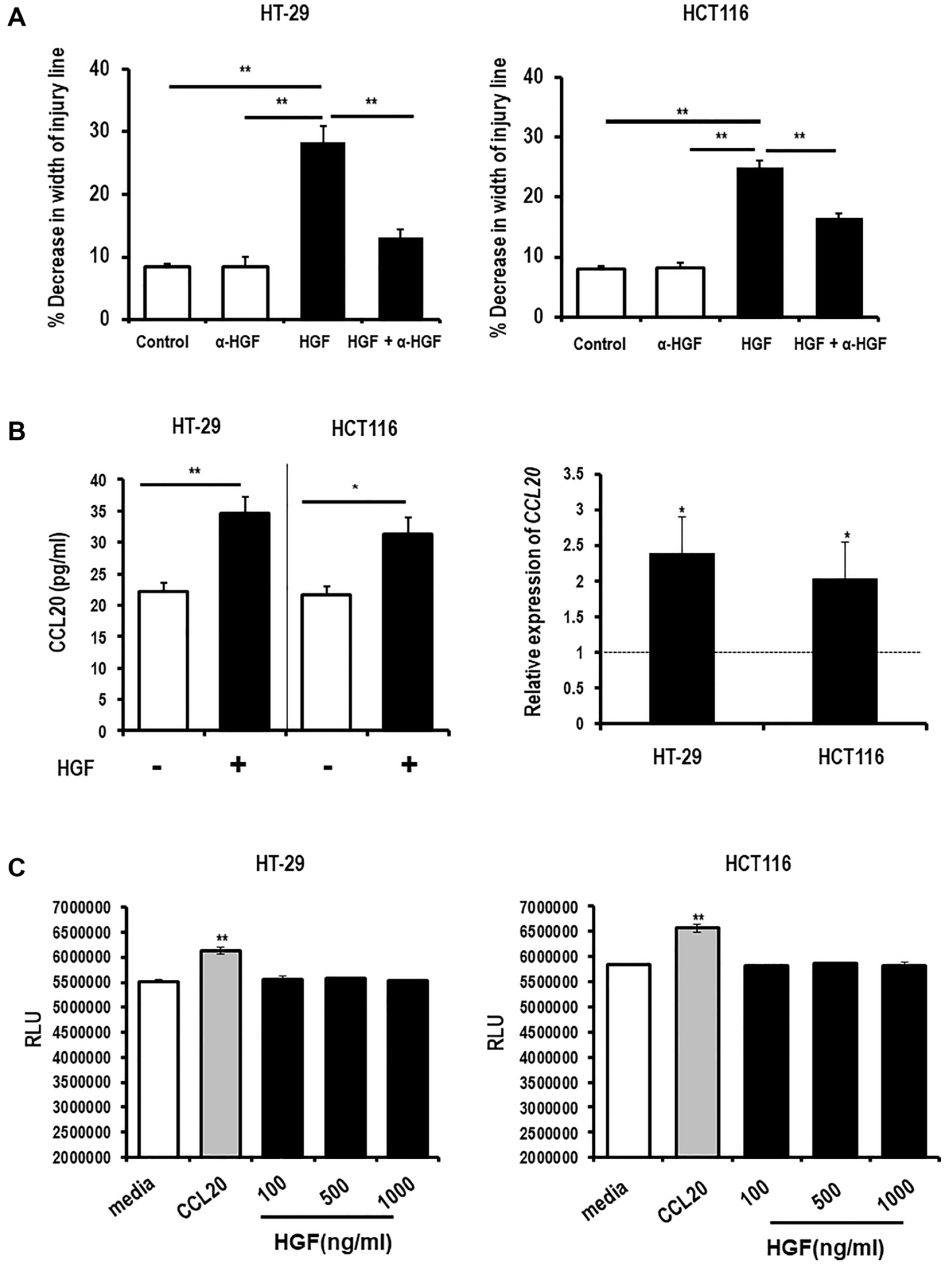
HGF induces colorectal cancer cell migration and CCL20 production but not proliferation. (**A**) Migration of the colorectal cancer cell lines HT-29 (left) and HCT116 (right) was measured after exposure to HGF (5 ng/ml) and an anti-HGF antibody (α-HGF, 10 μg/ml) for 48 hours using the wound healing assay. (**B**) CCL20 production by colorectal cancer cells after exposure to HGF and α-HGF for 48 hours was measured in bulk culture supernatants by ELISA (left, and in mRNA of cell lysates by qRT-PCR (right). (**C**) Proliferation of colorectal cancer cells after exposure to CCL20 (100 ng/ml) or HGF at the concentrations shown for 48 hours was measured by the CellTiter-Glo assay. RLU = relative light units. ^*^represents *p* < 0.05, ^**^represents *p* < .01, and ^***^represents *p* < 0.001.

We next explored whether the HGF secretion induced by CCL20 stimulation of colon cancer epithelial cells is relevant to the functional effects of CCL20. HGF is known to be involved in migration of many cancer cell types, and a few reports have indicated that it may induce CCL20 secretion. We first checked if HGF could induce colorectal cancer cell migration, CCL20 secretion, proliferation. HGF was found to induce migration of both the colorectal cancer cell lines using the wound healing assay (Figure 2A, Supplementary Figure 3A). We then assessed if HGF induces secretion of CCL20, and stimulation with HGF was found to induce CCL20 production at both the protein and mRNA levels in both cell lines (Figure 2B). Surprisingly, stimulation with HGF did not increase colorectal cancer cell proliferation at doses up to 1000 ng/ml (Figure 2C).

### CCL20-dependent colorectal cancer cell migration and CCL20 production, but not proliferation, are mediated through HGF

**Figure 3:**
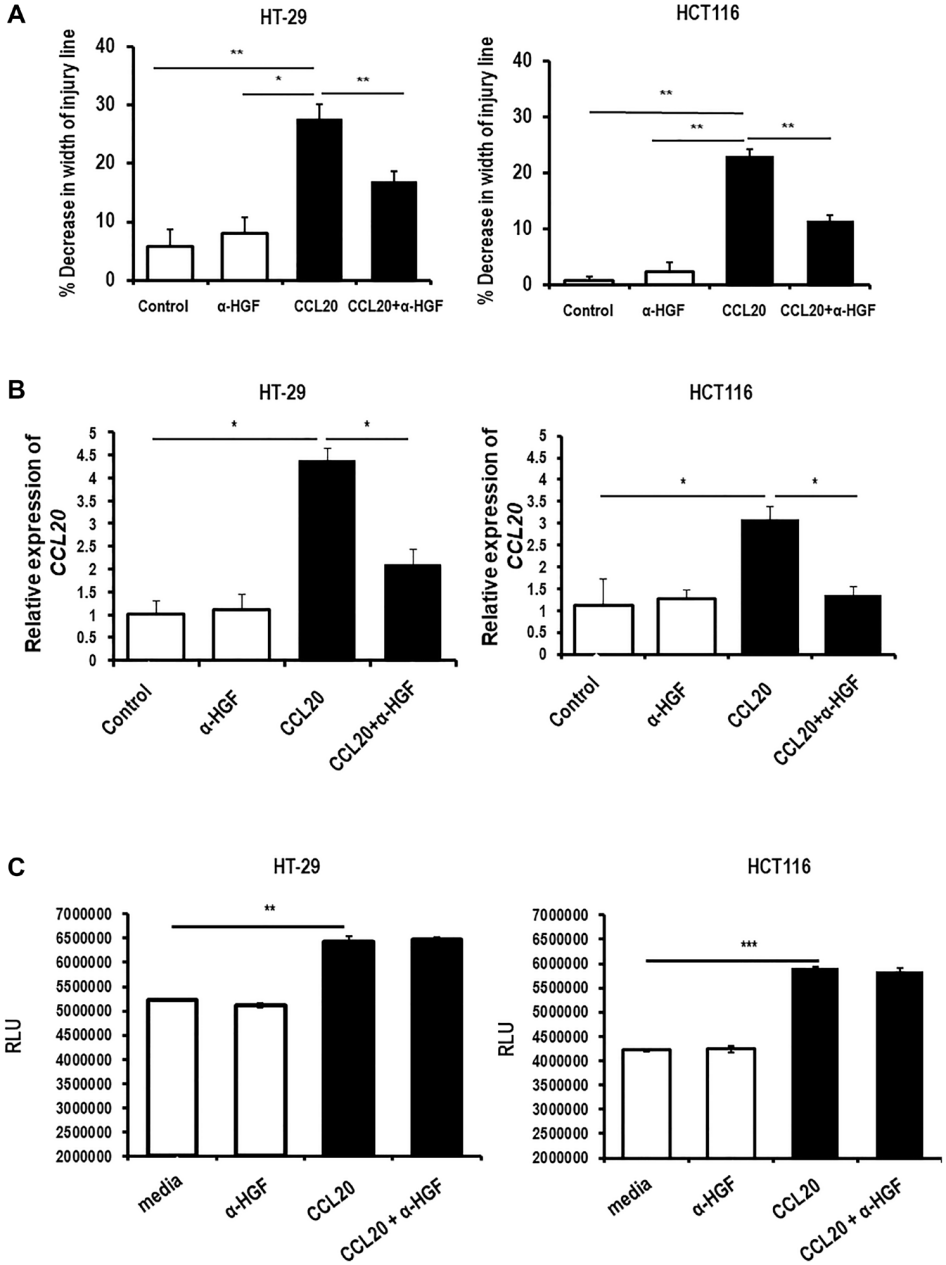
CCL20-dependent colorectal cancer cell migration and CCL20 production, but not proliferation, are mediated by HGF. (**A**) Migration of the colorectal cancer cell lines HT-29 (left) and HCT116 (right) was measured in the presence of CCL20 (100 ng/ml) and an anti-HGF antibody (α-HGF, 10 μg/ml) for 48 hours using the wound healing assay. (**B**) CCL20 production by colorectal cancer cells in the presence of CCL20 and α-HGF for 48 hours was measured in mRNA of cell lysates by qRT-PCR. (**C**) Proliferation of colorectal cancer cells in the presence of CCL20 and α-HGF for 48 hours was measured by the CellTiter-Glo assay. RLU = relative light units. ^*^represents *p* < 0.05, ^**^represents *p* < 0.01, and ^***^represents *p* < 0 001.

Having shown that CCL20 induces secretion of HGF by colorectal cancer cells and that HGF can induce colorectal cancer cell migration and CCL20 secretion, we next investigated whether CCL20-dependent colorectal cancer cell migration and CCL20 production are mediated through HGF. We demonstrated that the increased migration seen for both cell types after exposure to CCL20 was inhibited by an anti-HGF antibody (Figure 3A, Supplementary Figure 3B). Similarly, the production of *CCL20* induced by stimulating the colorectal cancer cells with CCL20 was abrogated by the anti-HGF antibody (4.4 fold for CCL20 versus 2.1 fold for CCL20 with anti-HGF antibody in HT-29 and 3.1 fold versus 1.4 in HCT116, Figure 3B). As, HGF did not induce colorectal cancer cell proliferation, inhibition of HGF also did not have any effect on the increased proliferation induced by CCL20 (Figure 3C).

We next assessed for a possible role of TGF-α or amphiregulin in CCL20-dependent colorectal cancer cell migration, CCL20 production, or proliferation as secretion of these factors in response to CCL20 was seen in some of our assays, and as a prior study showed that CCL20-dependent proliferation in the CaCo-2 colorectal cancer cells was dependent on amphiregulin secretion.[13] In contrast to what was seen for HGF, TGF-α and amphiregulin did not appear to play a role in CCL20-dependent colorectal cancer cell migration, CCL20 production, or proliferation. Administration of exogeneous TGF-α and amphiregulin did not induce migration of the colorectal cancer cell lines (Supplementary Figure 4A), while blocking TGF-α and amphiregulin action did not diminish the effects of CCL20 in inducing CCL20 secretion (Supplementary Figure 4B) or proliferation (Supplementary Figure 4C).

### CCL20 induces HGF-dependent ERK1/2 phosphorylation in neoplastic epithelial colorectal cancer cells

**Figure 4:**
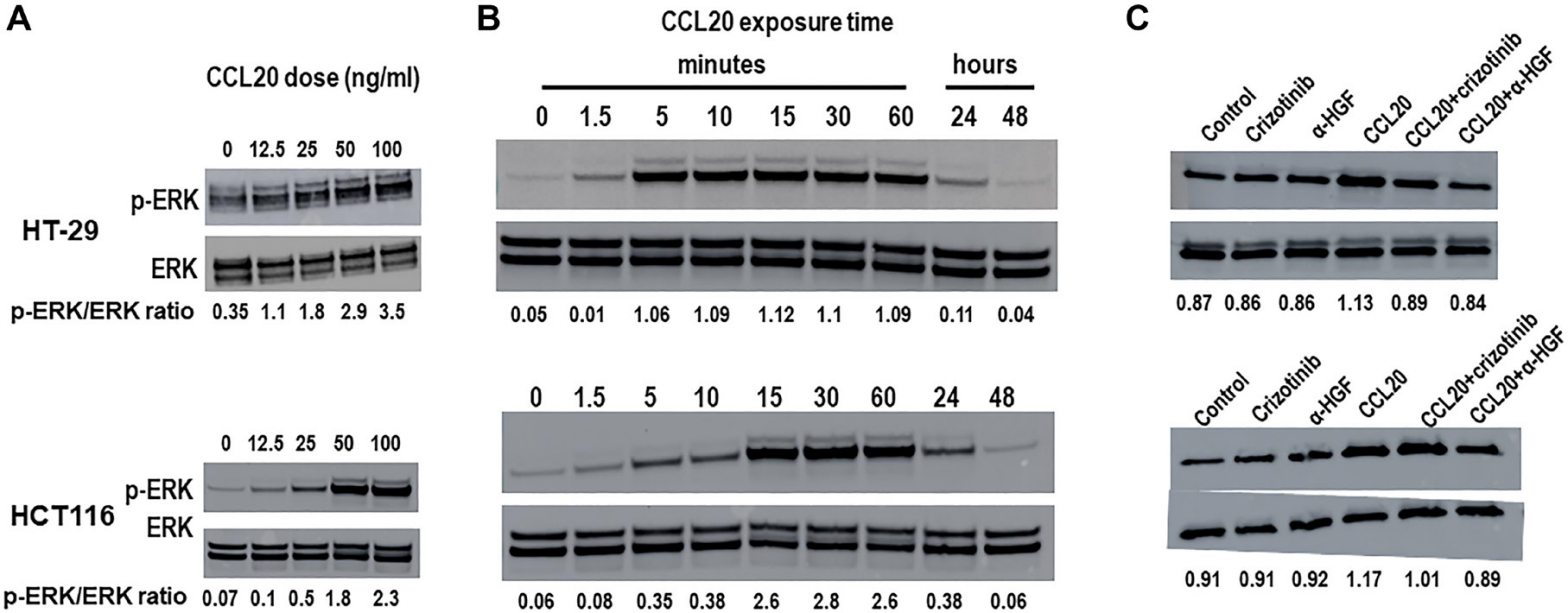
CCL20 induces HGF-dependent ERK phosphorylation in colorectal cancer cells. Levels of ERK and phosphorylated ERK (p-ERK) were measured in lysates of the colorectal cancer cell lines HT-29 (top) and HCT116 (bottom) after stimulation with CCL20 at (**A**) various concentrations for 10 minutes and (**B**) various time points with 100 ng/ml CCL20 using Western blots. (**C**) Levels of ERK and phosphorylated ERK were measured in colorectal cancer cells after exposure to crizotinib (5 μM), CCL20 (100 ng/ml), and an anti-HGF antibody (α-HGF, 10 μg/ml) for 10 minutes.

Several growth factors, including HGF signal through the MEK-ERK pathway. Thus, we next investigated whether the key molecules in this pathway, extracellular signal-regulated kinases (ERK) also known as mitogen-activated protein kinases (MAPK), mediate CCL20-dependent colorectal cancer migration, CCL20 secretion, and proliferation. We first assessed expression of ERK1/2 and phosphorylated ERK1/2 after stimulation of the colorectal cancer cell lines with CCL20. We observed that CCL20 stimulation induced ERK1/2 phosphorylation in both a concentration-dependent (Figure 4A) and a time-dependent (Figure 4B) manner. Moreover, CCL20-mediated ERK1/2 phosphorylation was inhibited by an anti-HGF antibody (Figure 4C), implying that CCL20-dependent ERK signaling in colorectal cancer cells is mediated through HGF.

### CCL20-dependent colorectal cancer cell migration and CCL20 production are mediated by ERK1/2

**Figure 5:**
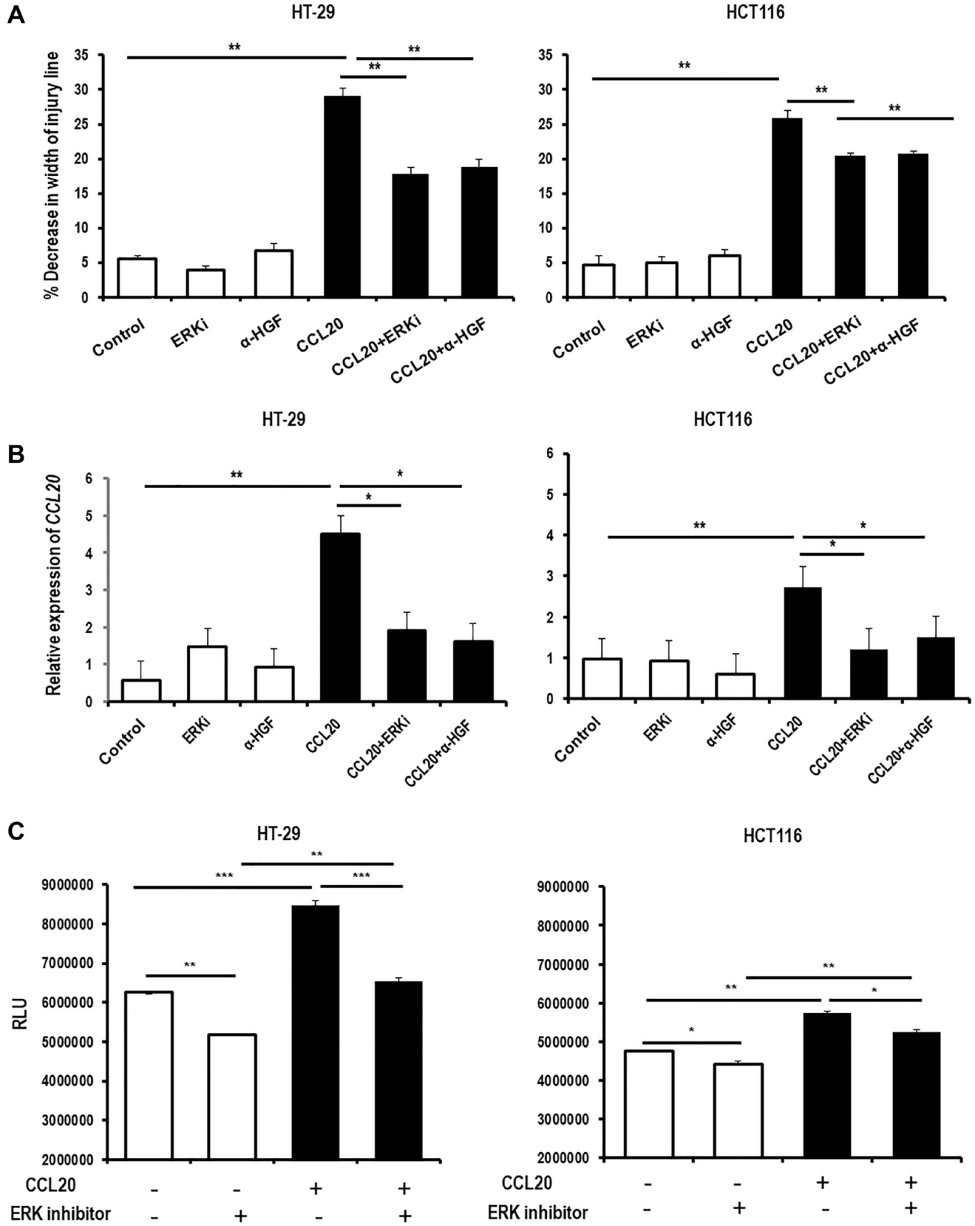
CCL20-dependent colorectal cancer cell migration and CCL20 production are mediated by ERK, while proliferation is mediated by ERK through a CCL20-independent process. (**A**) Migration of the colorectal cancer cell lines HT-29 (left) and HCT116 (right) was measured in the presence of CCL20 (100 ng/ml), the ERK inhibitor SCH772984 (ERKi,100 nM), and an anti-HGF antibody (α-HGF, 10 μg/ml) for 48 hours using the wound healing assay. (**B**) CCL20 production by colorectal cancer cells in the presence of CCL20, the ERK inhibitor (ERKi), and α-HGF was measured in mRNA of cell lysates by qRT-PCR. (**C**) Proliferation of colorectal cancer cells in the presence of CCL20 and the ERK inhibitor for 48 hours was measured by the CellTiter-Glo assay. RLU = relative light units. ^*^represents *p* < 0.05, ^**^represents *p* < 0.01, and ^***^represents *p* < 0.001.

We next checked if the ERK1/2 activation in colorectal cancer cells caused by CCL20 stimulation is required for CCL20-dependent migration. Treating the colorectal cancer cells with an ERK1/2 inhibitor during CCL20 stimulation blocked CCL20-induced migration in both colorectal cancer cell lines to the extent seen with inhibition of HGF using the anti-HGF antibody (Figure 5A). Similarly, ERK1/2 inhibition attenuated CCL20-dependent *CCL20* mRNA and CCL20 protein production by the colorectal cancer cells to the degree seen with HGF blockade (4.5 fold for CCL20 versus 1.9 fold for CCL20 with ERK1/2 inhibitor and 1.6 for CCL20 with anti-HGF antibody in HT-29, 2.7 fold versus 1.2 fold and 1.5 fold in HCT16, Figure 5B, Supplementary Figure 5). Interestingly, ERK1/2 inactivation reduced the level of proliferation seen in the colorectal cancer cells both in the presence and the absence of CCL20 (Figure 5C) indicating that proliferation of colorectal cancer cells is mediated by ERK1/2 through a CCL20-independent process.

### CCL20-dependent proliferation is mediated by MSP

**Figure 6:**
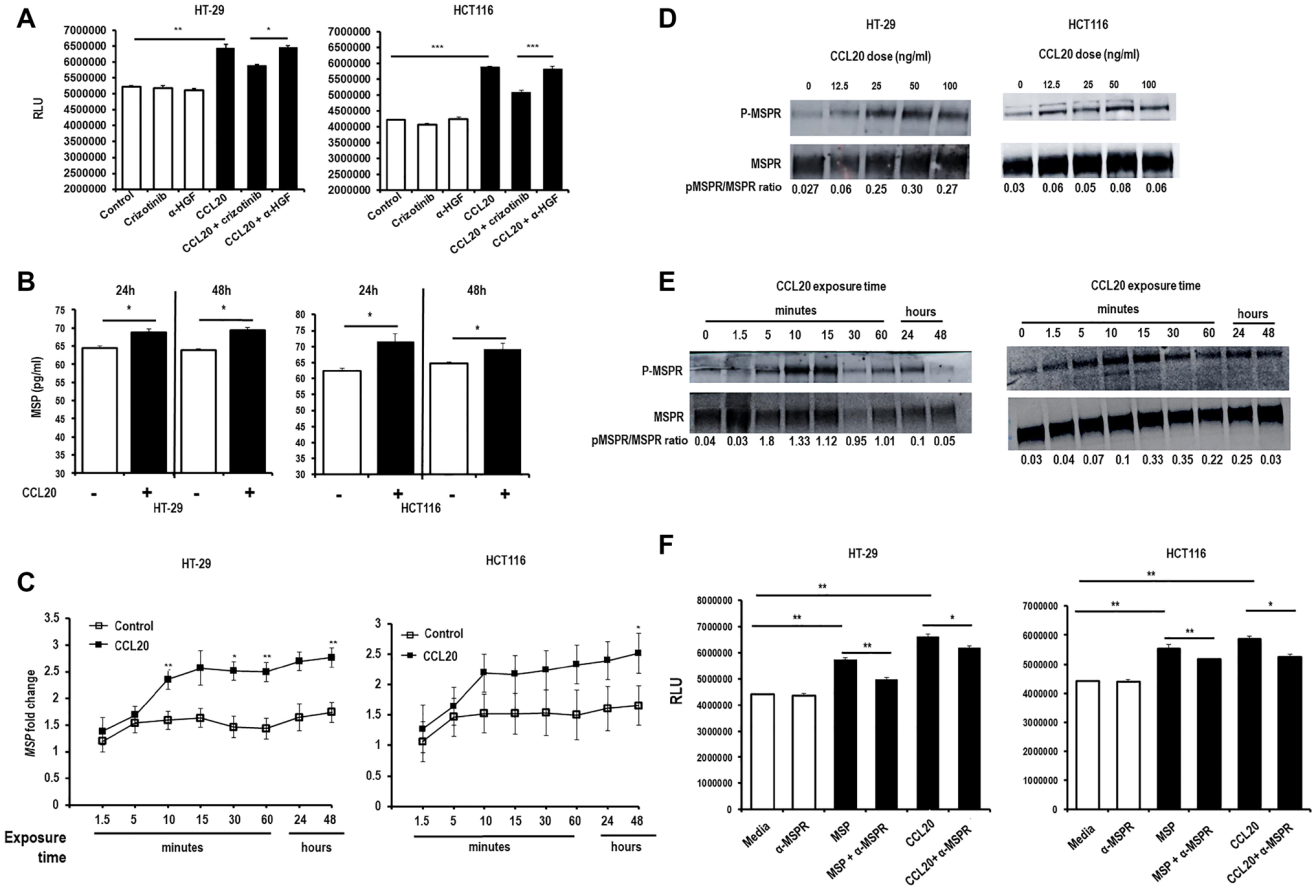
CCL20-dependent colorectal cancer cell proliferation is mediated by MSP. (**A**) Proliferation of the colorectal cancer cell lines HT29 (left) and HCT116 (right) was measured in the presence of crizotinib (5 μM), an anti-HGF antibody (α-HGF, 10 μg/ml), and CCL20 (100 ng/ml) for 48 hours using the CellTiter-Glo assay. RLU = relative light units. (**B**) MSP secretion was measured by ELISA after stimulating colorectal cancer cells with CCL20 for 24 and 48 hours. (**C**) Production of *MSP* was assessed in mRNA of colorectal cell lysates by qRT-PCR at various time points after exposure to CCL20. Phosphorylation of the MSP receptor MSPR after stimulation with CCL20 was measured by Western blots of lysates from colorectal cancer cells at various (**D**) concentrations for 10 minutes and (**E**) time points with 100 ng/ml CCL20. (**F**) Proliferation of colorectal cancer cells after exposure to an anti-MSPR antibody (α-MSPR, 10 μg/ml), MSP (100 ng/ml), and CCL20 for 48 hours was measured by the CellTiter-Glo assay. ^*^represents *p* < 0.05, ^**^represents *p* < 0.01, and ^***^represents *p* < 0.001.

While CCL20-dependent proliferation was not affected by the administration of a blocking antibody specifically targeting HGF, we found that the administration of crizotinib, a small molecular tyrosine kinase inhibitor that blocks signaling through the HGF receptor c-Met diminished CCL20-mediated proliferation (Figure 6A). As tyrosine kinase receptors are well known for their lack of specificity, we hypothesized that this effect may be mediated by the blockade by crizotinib of a tyrosine kinase receptor other than c-Met. Crizotinib is known to inhibit the homologous receptor macrophage-stimulating protein receptor (MSPR), also known as recepteur d’origine nantais (RON) [19]. We thus investigated whether MSPR and its ligand macrophage-stimulating protein (MSP), also known as hepatocyte growth factor-like protein, mediate CCL20-dependent proliferation. In fact, CCL20 induced secretion of MSP protein (Figure 6B) and production of *MSP* mRNA (2.8 fold at 48 hours for HT-29 and 2.5 fold for HCT116, Figure 6C) by both cell lines. Furthermore, CCL20 stimulation was found to result in phosphorylation of MSPR in both the colorectal cancer cell lines in a concentration-dependent (Figure 6D) and time-dependent (Figure 6E) manner. Last, we found that MSP induced proliferation of the colorectal cancer cell lines, and that blocking MSP with a specific antibody inhibited the induced proliferation induced by CCL20 (Figure 6F).

## DISCUSSION

The importance of CCL20-CCR6 signaling in colorectal cancer has been demonstrated by several studies. CCL20-CCR6 interactions in colorectal cancer have been shown to modulate tumor stroma as well as to have direct effects on neoplastic epithelial cells. In particular, CCL20 acting on CCR6 expressed by colorectal cancer neoplastic epithelial cells induces proliferation [1, 8, 12–15], migration [12, 14–17], and initiates an auto-feedback loop by inducing further secretion of CCL20 [8]. The mechanisms through which CCL20-CCR6 interactions elicit these effects is poorly understood. This study aimed to elucidate these mechanisms.

In this study, we show that CCL20 stimulation induces hepatocyte growth factor (HGF) production and phosphorylation of its receptor c-Met by colon cancer neoplastic epithelial cells. We also observed that HGF induces colorectal cancer cell migration and CCL20 production. Similarly, we found that CCL20-dependent colorectal cancer cell migration and CCL20 production are mediated through HGF. We demonstrate that CCL20 induces HGF-dependent ERK1/2 phosphorylation in neoplastic epithelial colorectal cancer cells and that CCL20-dependent colorectal cancer cell migration and CCL20 production are mediated by ERK1/2. Last, we observed that CCL20-dependent proliferation is mediated by macrophage-stimulating protein (MSP) but not HGF.

HGF is a growth factor in the plasminogen-related growth factor family. HGF exerts its effects by binding to the receptor tyrosine kinase c-Met, which is encoded by the MET proto-oncogene. Colorectal cancer is known to be associated with oncogenic dysregulation of the HGF/c-Met pathway. Overexpression of both HGF and c-Met have been demonstrated in this disease [20]. Furthermore, the importance of this pathway is corroborated as overexpression of HGF and c-Met in the colorectal cancer microenvironment is associated with poor clinical outcome [20, 21]. Experimental evidence has shown that signaling through the HGF/c-Met pathway is associated with enhanced tumorigenicity, invasiveness and metastasis in several *in vitro* [22] and *in vivo* xenograft [23, 24] and transgenic mouse models [25, 26]. In fact, therapeutic targeting of HGF/c-Met signaling in the treatment of colorectal cancer is an active area of investigation with several compounds currently in human clinical trials including antibodies directed at HGF, antibodies directed at c-Met, and tyrosine kinase inhibitors with activity against c-Met.

To our knowledge, the findings that CCL20 induces secretion of HGF and phosphorylation of c-Met and that this pathway mediates the effects of CCL20 in eliciting migration and further secretion of CCL20 by colorectal cancer cells have not previously been reported in the literature. Our finding that signaling through the HGF/c-Met pathway induces migration of colorectal cancer cells is substantiated by several studies including those using the HT-29 and HCT116 cell lines [27–29]. In fact, the HGF/c-Met pathway has been implicated in the metastatic process of colorectal cancer [30–32]. Similarly, while it has not been previously reported that CCL20 induces HGF secretion or that HGF is involved in CCL20-mediated CCL20 secretion, other studies have found a link between HGF and secretion of CCL20. HGF was shown to increase expression of CCL20 in neurons [33], while in cancer, HGF stimulation induced CCL20 secretion in papillary carcinoma of the thyroid [34] and inhibition of HGF resulted in lower CCL20 levels in a renal cancer model [35].

There are mixed reports on the role of HGF in inducing proliferation in colorectal cancer cells including the HT-29 and HCT116 cell lines [27, 36–38]. One study found that HGF induces a nominal increase in proliferation of HT-29 and HCT116 [38]. Another report found HGF to induce proliferation in HCT116 but not HT-29 [37]. Yet another study found HGF to inhibit the proliferation of HT-29 [27]. Overall, these data, including our own findings, appear to suggest that HGF is not a powerful mitogenic factor for colorectal cancer cells, particularly the HT-29 and HCT116 cell lines. The varying results between studies may have arisen due to subtle differences in cultural conditions and genetic changes that might have arisen during culture.

Our finding that CCL20-CCR6 interactions signal through the ERK/MAPK pathway is supported by studies involving various cancer types including colorectal cancer. It has been demonstrated that increased expression of CCL20 is associated with increased expression of p-ERK in human breast cancer; melanoma; and head and neck squamous cell carcinoma tumor tissue [39]. CCL20 stimulation has been demonstrated to directly cause phosphorylation of ERK in colorectal cancer cells in several studies, although the importance of ERK in mediating the downstream effects of CCL20 was not shown [3, 12, 13]. Notably, one study showed that CCL20-induced ERK phosphorylation in the Caco-2 cell line was mediated through a pathway involving the growth factor amphiregulin, the epithelial growth factor receptor (EGFR), and matrix metalloproteinase [13]. In lung cancer, CCL20-mediated MEK signaling was shown to mediate proliferation [40] and migration [40], while in breast cancer CCL20-mediated MEK signaling was shown to mediate proliferation [41]. MEK is the kinase immediately upstream of ERK that is responsible for phosphorylating ERK in the Ras-Raf-MEK-ERK signaling pathway.

MSP, also known as hepatocyte growth factor-like protein, is a growth factor with 45% homology to HGF [42]. Similar to HGF, MSP mediates a variety of cellular effects in normal tissues including proliferation, migration and differentiation [43, 44]. The receptor for MSP, macrophage-stimulating protein receptor (MSPR), also known as recepteur d’origine nantais (RON), is a receptor tyrosine kinase in the Met proto-oncogene family with very similar functional domains to c-Met having 63% homology in the extracellular domain and 25% homology in the tyrosine kinase domain [45]. The importance of MSP-MSPR signaling in colorectal cancer is substantiated by several lines of evidence. Overexpression of MSPR is often seen in human colorectal cancer [46–52]. MSP overexpression has been associated with poor prognosis in colorectal cancer [48, 50, 52, 53]. Furthermore, MSP-MSPR signaling has been associated with colorectal cancer growth and invasion *in vitro* as well as tumor growth *in vivo* [49, 52, 54]. Our finding that MSP induces proliferation in colorectal cancer cells is supported by other studies [49, 52, 54]. However, to our knowledge, an association between CCL20 and the MSP-MSPR pathway or a role of this pathway in mediating CCL20-dependent proliferation has not previously been shown.

It bears mentioning that a prior report implicated the involvement of an autocrine pathway in CCL20-CCR6 signaling in colorectal cancer cells reliant on a secreted factor other than the pathways involving HGF and MSP shown in this study [55]. This study showed that CCL20-dependent proliferation of colorectal cancer cells was dependent on amphiregulin secretion and EGFR phosphorylation through an ERK-mediated process. These findings were only shown in the Caco-2 cell line. While we did find significantly increased amphiregulin secretion by the HT-29 cell lines at 48 hours after CCL20 exposure, we did not find this at 6 hours or at either time point in the HCT116 cell line. Also, we did not find significantly increased expression of *amphiregulin* message at any time point in either cell line. Last, blockade of amphiregulin did not abrogate CCL20-mediated proliferation in either cell line. These discrepancies may relate to the different cell lines used. Further studies will be needed to determine whether the growth factor dependence of CCL20-CCR6 signaling varies in different contexts.

There are other limitations of this study. Our experiments were limited to the colon cancer cell lines HT-29 and HCT116, and thus may not be reflective of all colorectal cancer cells. The pathways involved in CCL20-CCR6 signaling may relate to genetic and epigenetic events within the tumor. It is now recognized that the cellular phenotype and tumor behavior of colorectal cancers can be at least partially explained by the consensus molecular subtype (CMS) of each tumor, defined by global gene expression within that tumor. The CMS, in turn, is associated with the genetic mutations of the tumor acquired in oncogenesis [56]. The HT-29 cell line has been associated with the CMS 3/metabolic subtype and is known to be BRAF mutant, microsatellite stable (MSS), and to be negative for the CpG island methylator phenotype (CIMP) [57]. In contrast, HCT116 has been associated with the CMS 4/mesenchymal subtype, is KRAS mutant, has microsatellite instability (MSI), and is CIMP+ [57]. The Caco-2 cell line, which has been shown to possibly have a differing CCL20-CCR6 signaling pathway in a prior study [13], is associated with the CMS4/mesenchymal subtype, but is KRAS/BRAF wild type, MSS, and CIMP [57]. Another limitation of this study is that all of our experiments were performed *in vitro* using two-dimensional culture, so they do not shed light on how the tumor microenvironment may influence the effect of CCL20-CCR6 signaling within colorectal tumors. In fact, in colorectal cancer, HGF has been reported to elicit its effects through secretion by stromal cells such as fibroblasts and cancer-associated fibroblast, which then activates c-Met on adjacent colon cancer epithelial cells in a paracrine fashion [58–60]. Furthermore, c-Met and MSPR have been shown to elicit cross-talk with HGF-c-Met signaling resulting in MSPR tranphosphorylation and MSP-MSPR signaling resulting in c-Met transphosphorylation [61, 62]. Last, it is possible that the dose of CCL20 used in this study was not physiologically relevant. We chose the dose of 100 ng/ml as doses of 100 ng/ml or greater are most commonly used in *in vitro* studies of colorectal cancer [3, 12, 13, 17] and other malignancies [39, 40, 63].

In summary, we have demonstrated that CCL20 induces proliferation, migration and further secretion of CCL20 by neoplastic colorectal cancer cells through pathways mediated by autocrine secretion of growth factors. CCL20-mediated migration and CCL20 secretion were shown to be regulated through a pathway involving HGF, its receptor c-Met, and ERK phosphorylation. In contrast, CCL20-mediated proliferation was demonstrated to be independent of HGF/c-Met signaling, but was instead regulated through MSP and its receptor MSPR. Further studies are necessary to elucidate the role of these signaling pathways *in vivo* as the tumor microenvironment may be involved in the production and/or the effects of CCL20, HGF and MSP through cells such as macrophages, cancer-associated fibroblasts, and myofibroblasts. Nevertheless, these studies broaden the therapeutic options for targeting important cancer-promoting pathways in colorectal cancer. In particular, upregulation of HGF or MSP showed be assessed as potential resistance pathways to CCL20-CCR6 targeting in colorectal cancer, and similarly, the efficacy of combinatorial approaches of blocking either or both the HGF-c-Met pathway or the MSP-MSPR pathway along with the CCL20-CCR6 pathway should be sought.

## MATERIALS AND METHODS

### Cell lines and reagents

The human colon cancer cell lines HT-29 and HCT116 were purchased from American Type Culture Collection (ATCC; Manassas, VA, USA). The cell lines were expanded in a short culture to make frozen stock vials. A new stock vial was opened and cultured for each experiment. Cell lines were grown in RPMI 1640 (Life Technologies, Carlsbad, CA, USA) with 10% fetal bovine serum (FBS) (Life Technologies, USA). All cytokines, growth factors, and inhibitors used in cell culture experiments are listed in Supplementary Table 1.

### ELISA

Human cancer cell lines were cultured in RPMI without FBS with indicated growth factors and inhibitors for desired time. Culture supernatants were collected and analyzed using ELISA kits (R&D Systems, Minneapolis, MN) following the manufacturer’s protocol. In experiments analyzing time points < 24 hours, fresh media (including growth factor) was replaced 2 hours prior to collection when supernatants were collected after 24 hours of overnight culture. For the timepoint ≥ 24 hours media was not replaced.

### qRT-PCR

The RNeasy Plus Universal Kit (Qiagen, Hilden, Germany) was used for total RNA extraction following the instructions in the manufacturer’s protocol. Briefly, 2 × 10^5^ human colon cancer cells were mixed with 1 mL of the QIAzol Lysis Reagent. 1 μg of total RNA was reverse transcribed using the SuperScript III First-Strand Synthesis System (Life Technologies, USA) where oligo-dT was used as first synthesis primer to make cDNA. qRT-PCR was then performed on the cDNA. The primer sequences used are provided in Supplementary Table 2. 2 μL of cDNA and 0.5 μM of each primer were mixed with 10 μL of 2x Power SYBR Green PCR Master Mix (Life Technologies, USA) to a final reaction volume of 20 μL. All reactions were run in triplicate in 96-well optical reaction plates (Life Technologies, USA) using the ABI PRISM 7900HT Sequence Detection System (Life Technologies, USA) with the following conditions: 95°C for 10 minutes for initial melting followed by 40 cycles of 95°C melting for 10 seconds and 60°C annealing and extension for 1 minute. Relative expression was normalized to the housekeeping gene glyceraldehyde 3-phosphate dehydrogenase (*GAPDH)* and calculated using the 2^ΔΔCt^ method.

### Expression data from The Cancer Genome Atlas

Data from TCGA for colon adenocarcinoma (COAD) was downloaded using RTCGAToolbox [64] for further analysis. Correlation co-efficient and *p* values for Spearman and Pearson correlations between log2 scaled RSEM values for each gene pairs were calculated using cBioPortal [65].

### Western blots

Cells were lysed by treating with RIPA lysis buffer (Life Technologies, USA). Cell lysates were run in precast SDS-PAGE 4–15% gels (Novagen, Madison, WI, USA) and blotted on nitrocellulose membranes (Novagen). Membranes were washed with Tris-buffered saline (TBS) and blocked with fat-free milk in the same buffer for 1 hour at room temperature. The membranes were treated overnight at 4°C with primary antibodies directed against proteins or phospho-proteins of interest at dilutions as recommended by the manufacturers as listed in Supplementary Table 3. The next day, the membranes were washed and incubated with horseradish peroxidase (HRP) conjugated secondary antibodies at the recommended dilutions as described in Supplementary Table 3. Finally, the membranes were developed by using the Amersham ECL Plus kit (GE Healthcare, Piscataway, NJ, USA) per the manufacturer’s protocol.

### Scratch wound healing assay

5 × 10^4^ human colorectal cancer cells were seeded in 24-well plates and allowed to incubate for 24 hours in RPMI media with 10% FBS to form a confluent monolayer. Next, the media was replaced with RPMI without FBS and the confluent cells were wounded by scratching the culture well surface with a 100-μl pipette tip. Immediately following this, CCL20, HGF and/or inhibitors were added to the culture. The cultures were then left to heal the wound for 48 hours. The scratched wells were photographed by microscope at 0 and 48 hours, and distances were measured between the edges of the scratch. The percent decrease in the width of the scratch wound at 48 hours with respect to 0 hours was calculated.

### Cell proliferation assay

The CellTiter-Glo Luminescent Cell Viability Assay Kit (Promega, Madison, WI, USA) was utilized following instructions in the manufacturer’s protocol. Briefly, 5 × 10^3^ HT-29 or HCT116 cells were seeded in 100 μL of RPMI media without FBS in 96-well cell culture plates overnight. The next day, cytokines or growth factors were added to the culture medium at the desired concentrations in the presence or absence of inhibitors as indicated in the results, and the cultures were continued for 48 hours. The CellTiter-Glo Substrate and CellTiter-Glo buffer were mixed to make the CellTiter-Glo reagent. 100 μL of this reagent was mixed with the cultured cells for 2 minutes and kept at room temperature for another 10 minutes. Finally, a luminometer (FlexStation 3, Molecular Devices, San Jose, CA, USA) was used to measure luminescence.

### Statistics

Differences between means were determined by using the two-tailed Student’s unpaired *t*-test. For qRT-PCR experiments, the two-tailed Student’s unpaired *t*-test of ΔCt values was utilized. The coefficient of determination (R^2^) was calculated using the linear regression method. *p* values ≤ 0.05 were considered significant.

## SUPPLEMENTARY MATERIALS



## Abbreviations

CCR6: C-C chemokine receptor 6
CCL20: C-C chemokine ligand 20
COAD: colon adenocarcinoma
CIMP: CpG island methylator phenotype
CMS: consensus molecular subtype
EGF: epidermal growth factor
EGFR: epidermal growth factor receptor
ELISA: enzyme-linked immunosorbent assay
ERK: extracellular signal-regulated kinases
FBS: fetal bovine serum
FGF: fibroblast growth factor
GAPDH: glyceraldehyde-3-phosphate dehydrogenase
HGF: hepatocyte growth factor
c-Met: cellular mesenchymal to epithelial transition
IGF: insulin-like growth factor
MSP: macrophage stimulating protein
MSPR: macrophage stimulating protein receptor
LARC: liver activation regulated chemokine
MIP-3 α: macrophage inflammatory protein-3 alpha
MSI: microsatellite instability
MSS: microsatellite stable
MEK: mitogen-activated protein kinase kinase
PDGF: platelet derived growth factor
qRT-PCR: quantitative reverse transcriptase polymerase chain reaction
Ras: rat sarcoma
Raf: rapidly accelerated fibrosarcoma
RNA: ribonucleic acid
RON: recepteur d’origine nantais
RPMI: Roswell Park Memorial Institute medium
RSEM: RNA-Seq by expectation-maximization
SDS-PAGE: sodium dodecyl sulfate polyacrylamide gel electrophoresis
TCGA: The Cancer Genome Atlas
TGF-α: transforming growth factor-alpha
Treg: regulatory T cell
