# Supplementary material for: CCL20 induces colorectal cancer neoplastic epithelial cell proliferation, migration, and further CCL20 production through autocrine HGF-c-Met and MSP-MSPR signaling pathways
Source: Oncotarget. 2021 Nov 23;12(24):2323–37. doi: 10.18632/oncotarget.28131 (PMC8629403; doi:10.18632/oncotarget.28131)
Supplement: Supplementary file 1 [file oncotarget-12-2323-s001.pdf]

CCL20 induces colorectal cancer neoplastic epithelial cell proliferation, migration, and further CCL20 production through autocrine HGF-c-Met and MSP-MSPR signaling pathways

SUPPLEMENTARY MATERIALS

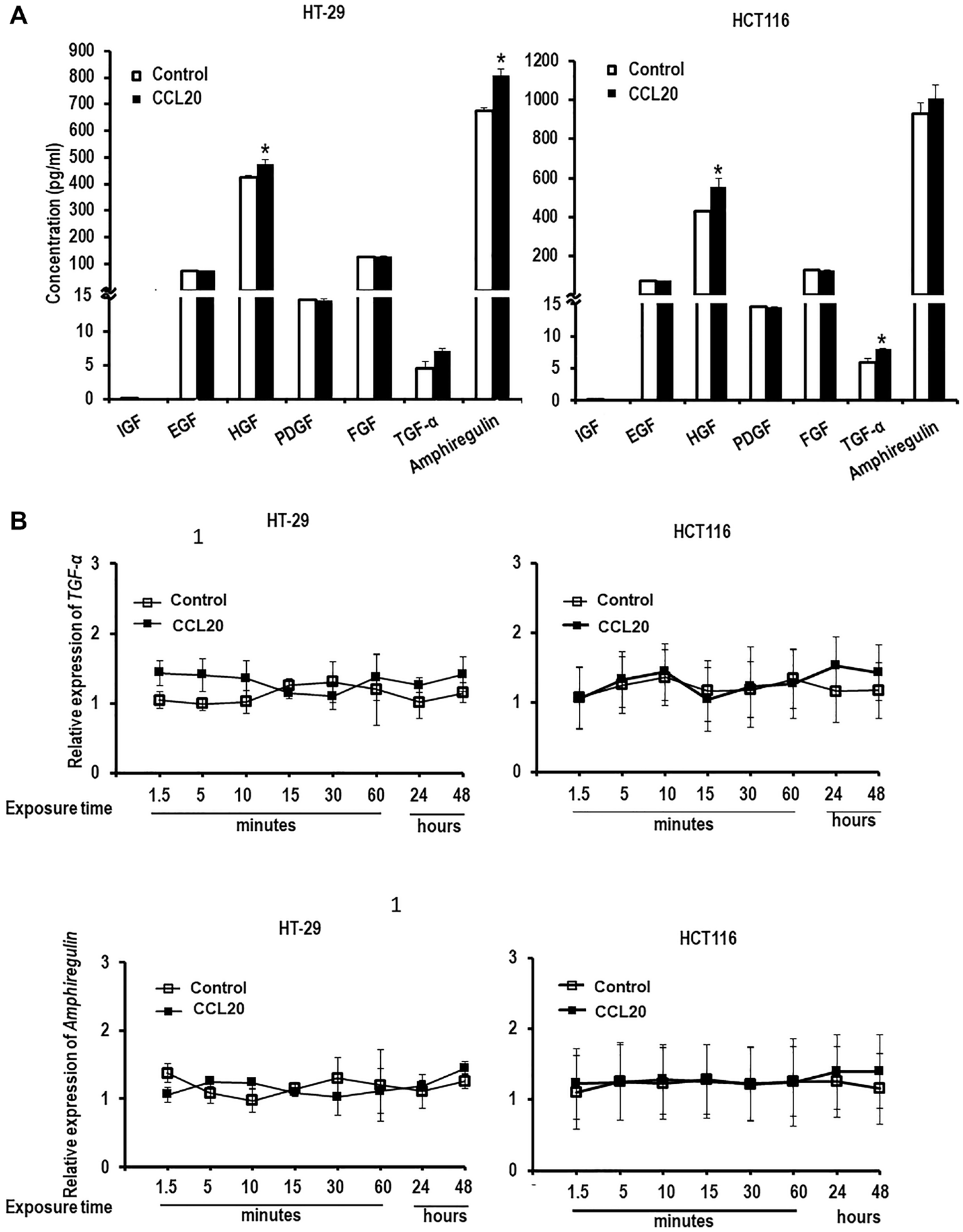

Supplementary Figure 1: CCL20 induces colorectal cancer cells to produce HGF. (**A**) Secretion of growth factors after stimulating the colorectal cancer cell lines HT-29 (left) and HCT116 (right) with CCL20 (100 ng/ml) for 48 hours was measured in bulk culture supernatants by ELISA. *represents $p < 0.05$. (**B**) Production of *TGF*-α and *amphiregulin* by colorectal cancer cells after stimulation with CCL20 was measured in mRNA of cell lysates by qRT-PCR at various time points.

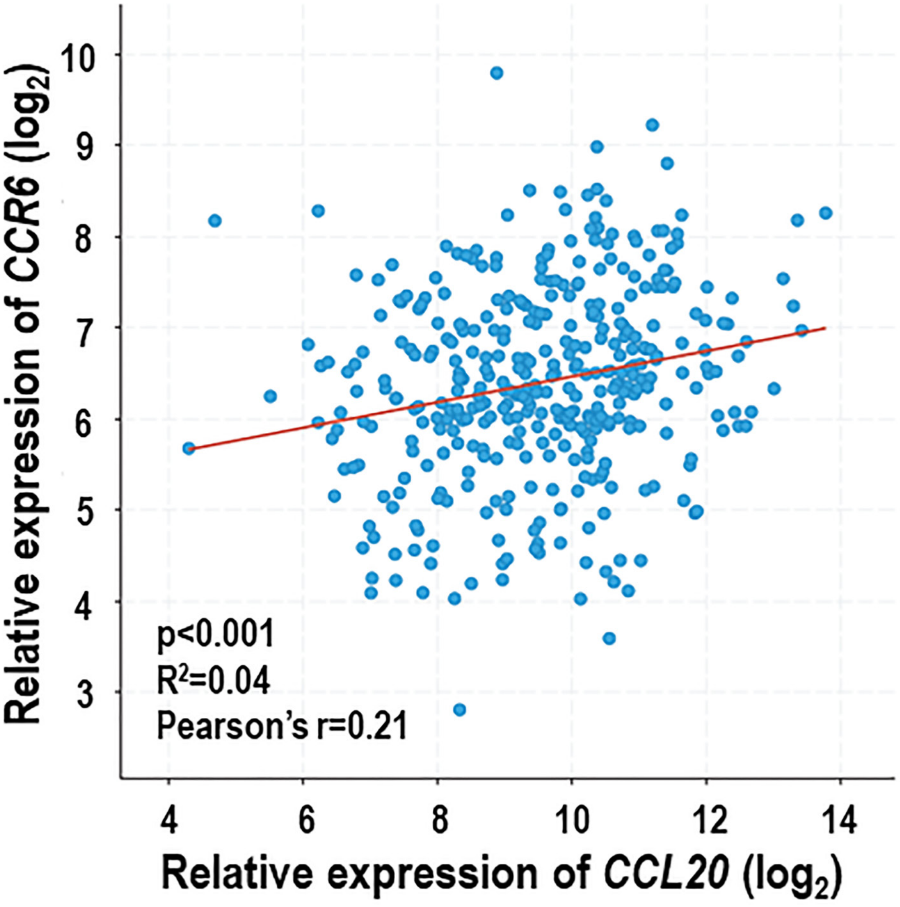

Supplementary Figure 2: Correlation between *CCL20* and *CCR6* expression in human colorectal cancer. Correlation was assessed using gene expression data from The Cancer Genome Atlas (TCGA).

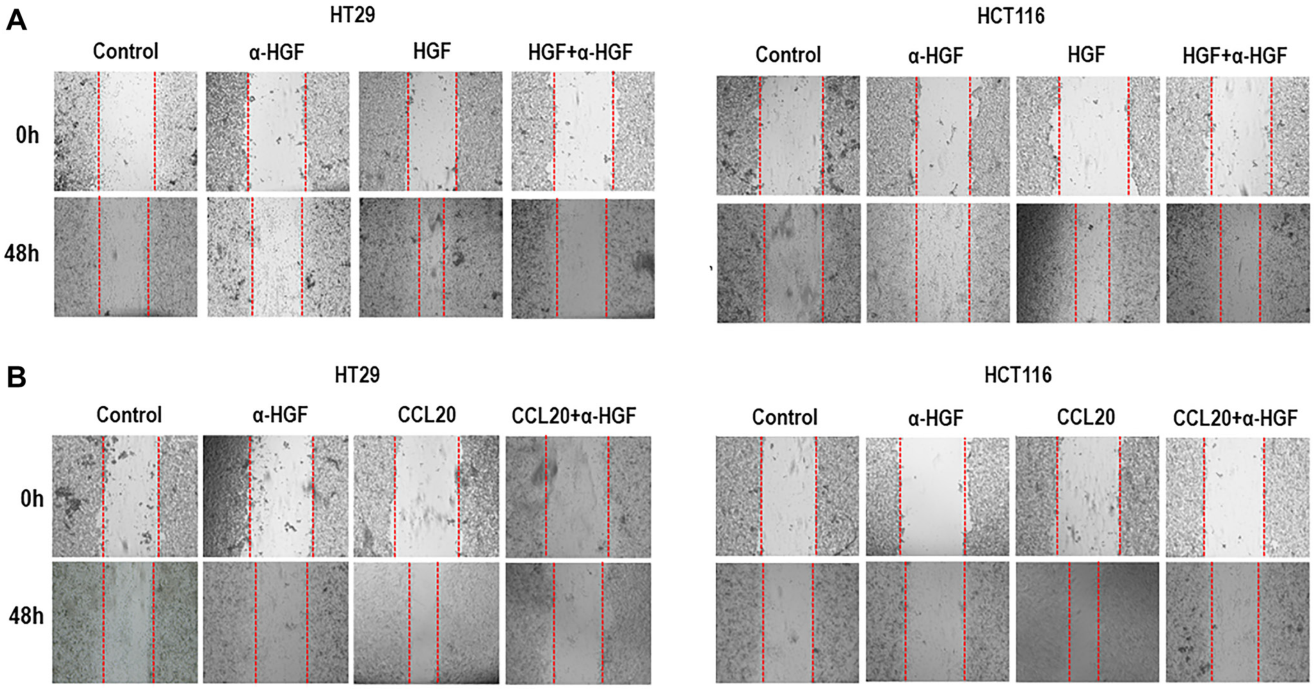

Supplementary Figure 3: HGF induces colorectal cancer cell migration, and CCL20-dependent colorectal cancer cell migration is mediated by HGF. Migration of the colorectal cancer cell lines HT-29 (left) and HCT116 (right) was measured after exposure to (**A**) HGF (5 ng/ml) and an anti-HGF antibody (α-HGF, 10 µg/ml) and (**B**) CCL20 (100 ng/ml) and an anti-HGF antibody using the wound healing assay by comparing the width of the scratch at the beginning of the assay (0 h) to that after 48 hours (48 h).

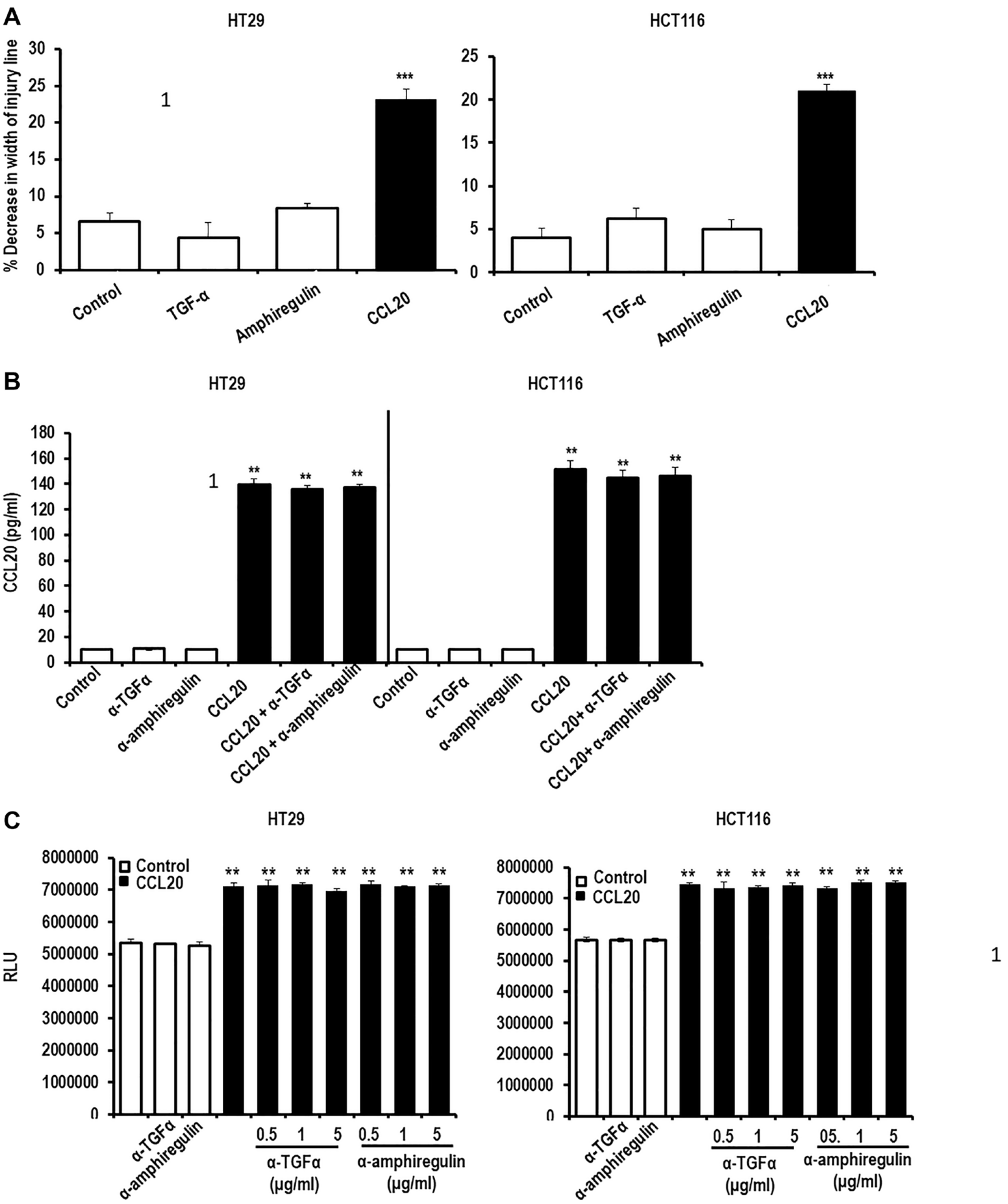

Supplementary Figure 4: CCL20-dependent colorectal cancer cell migration, CCL20 production, and proliferation are not mediated by TGF-α or amphiregulin. (**A**) Migration of the colorectal cancer cell lines HT-29 (left) and HCT116 (right) after stimulation with TGF-α (1 ng/ml), amphiregulin (30 ng/ml), or CCL20 (100 ng/ml) for 48 hours was assessed using the wound healing assay. (**B**) CCL20 production by colorectal cancer cells after exposure to an anti-TGF-α antibody (α-TGFα, 5 μg/ml), an anti-amphiregulin antibody (α-amphiregulin, 5 μg/ml), and CCL20 was measured bulk culture supernatants by ELISA at 48 hours. (**C**) Proliferation of cancer cells after exposure to α-TGFα (5 μg/ml unless specified), α-amphiregulin (5 μg/ml unless specified), and CCL20 for 48 hours was measured by the CellTiter-Glo assay. RLU = relative light units. **represents $p < 0.01$, and ***represents $p < 0.001$.

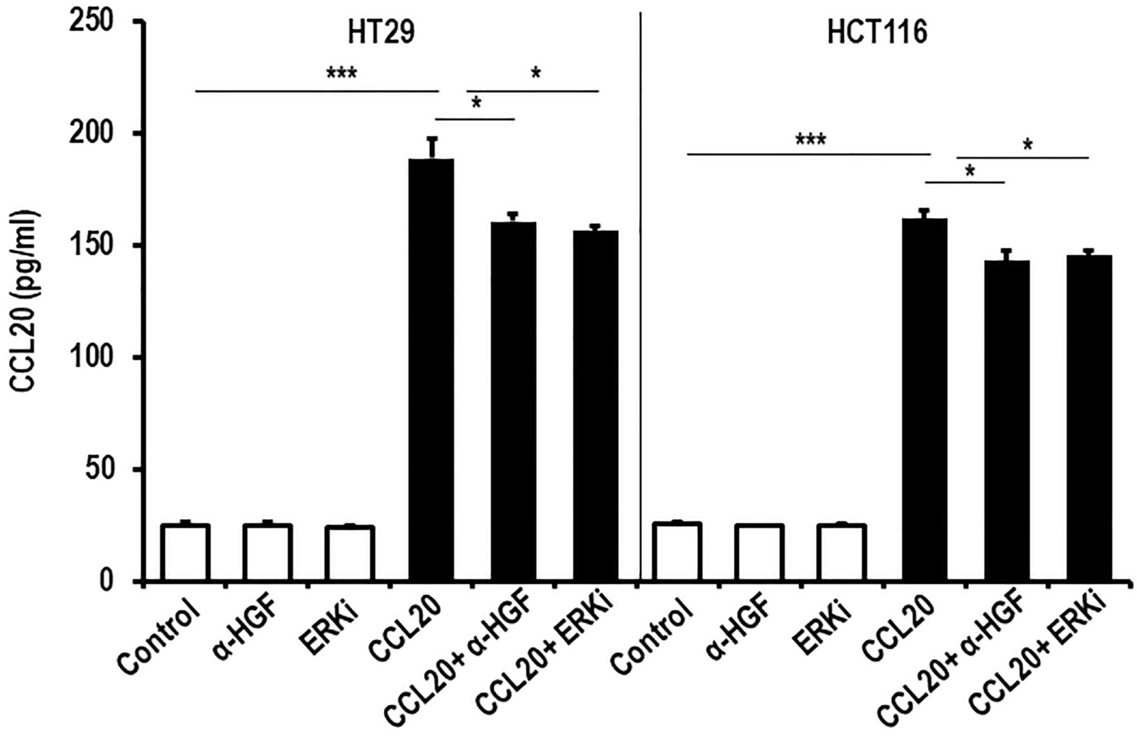

Supplementary Figure 5: CCL20-dependent CCL20 production by colorectal cancer cells is mediated by HGF and ERK. Secretion of CCL20 by the colorectal cancer cell lines HT-29 (left) and HCT116 (right) after exposure to an antibody against HGF (α-HGF, 10 µg/ml), an ERK inhibitor (SCH772984, 100 nM), and CCL20 (100 ng/ml) for 48 hours was measured in bulk culture supernatants by ELISA. *represents $p < 0.05$, **represents $p < 0.01$, and ***represents $p < 0.001$.

Supplementary Table 1: Cytokine, growth factors and inhibitors used

Reagents		Vendor (Location)
CCL20		R&D Systems, (Minneapolis, MN, USA)
HGF		R&D Systems, (Minneapolis, MN, USA)
TGFα		R&D Systems, (Minneapolis, MN, USA)
Amphiregulin		R&D Systems, (Minneapolis, MN, USA)
MSP		R&D Systems, (Minneapolis, MN, USA)
α-HGF	Goat polyclonal	R&D Systems, (Minneapolis, MN, USA)
α-MSP	Goat polyclonal	R&D Systems, (Minneapolis, MN, USA)
α-TGF	Goat polyclonal	R&D Systems, (Minneapolis, MN, USA)
α-amphiregullin	Mouse monoclonal	R&D Systems, (Minneapolis, MN, USA)
Crizotinib		Sigma-Aldrich, (St. Louis, MO, USA)
ERK inhibitor (SCH772984)		Selleck Chemicals (Radnor, PA, USA)

Supplementary Table 2: Primer sequences used in qRT-PCR

Gene	Sequence (5′ to 3′)	Reference (PMID)
CCL20 (forward)	CTGGCTGCTTTGATGTCAGT	[1]
CCL20 (reverse)	CGTGTGAAGCCCACAATAAA	[1]
HGF (forward)	GTAAATGGGATTCCAACACGAACAA	[2]
HGF (reverse)	TGTCGTGCAGTAAGAACCCAACTC	[2]
TGFα (forward)	GTAAAATGGTCCCCTCGGCT	[3]
TGFα (reverse)	GGGTCTGCACTCAGCGG	[3]
Amphiregulin (forward)	CTGTCGCTCTTGATACTCGG	[4]
Amphiregulin (reverse)	GCCAGGTATTTGTGGTTCGT	[4]
MSP (forward)	CCTCCCACATTCCGAAAACCA	[5]
MSP (reverse)	GCACTCCTGACAAATGGGTG	[5]
GAPDH (forward)	CAATGACCCCTTCATTGACC	[1]
GAPDH (reverse)	GACAAGCTTCCCGTTCTCAG	[1]

Supplementary Table 3: Antibodies used in western blot experiments

Antibody	Dilution	Host	Vendor (Location)
**Primary antibodies**			
Anti-human ERK	1:1000	Rabbit	Cell Signaling Technology, (Danvers, MA, USA)
Anti-human phospho-ERK	1:2000	Rabbit	Cell Signaling Technology, (Danvers, MA, USA)
Anti-human c-MET	1:1000	Rabbit	Cell Signaling Technology, (Danvers, MA, USA)
Anti-human phospho-c-MET	1:1000	Rabbit	Cell Signaling Technology, (Danvers, MA, USA)
Anti-human MSPR	1:200	Goat	R&D Systems, (Minneapolis, MN, USA)
Anti-human phospho-MSPR	1:200	Rabbit	R&D Systems, (Minneapolis, MN, USA)
**Secondary antibodies**			
Anti-rabbit IgG-HRP	1:5000	Goat	Cell Signaling Technology, (Danvers, MA, USA)
Anti-goat IgG-HRP	1:5000	Rabbit	Santa Cruz Biotechnology, (Dallas, TX, USA)

SUPPLEMENTARY REFERENCES

1. Kao CY, Huang F, Chen Y, Thai P, Wachi S, Kim C, Tam L, Wu R. Up-regulation of CC chemokine ligand 20 expression in human airway epithelium by IL-17 through a JAK-independent but MEK/NF-kappaB-dependent signaling pathway. J Immunol. 2005; 175:6676–85. https://doi.org/10.4049/jimmunol.175.10.6676. [PubMed]
2. Sakai K, Takeda M, Okamoto I, Nakagawa K, Nishio K. Multiple regulatory mechanisms of hepatocyte growth factor expression in malignant cells with a short poly(dA) sequence in the HGF gene promoter. Oncol Lett. 2015; 9:405–10. https://doi.org/10.3892/ol.2014.2702. [PubMed]
3. Kefaloyianni E, Muthu ML, Kaeppler J, Sun X, Sabbisetti V, Chalaris A, Rose-John S, Wong E, Sagi I, Waikar SS, Rennke H, Humphreys BD, Bonventre JV, et al. ADAM17 substrate release in proximal tubule drives kidney fibrosis. JCI Insight. 2016; 1. https://doi.org/10.1172/jci.insight.87023. [PubMed]
4. Latasa MU, Salis F, Urtasun R, Garcia-Irigoyen O, Elizalde M, Uriarte I, Santamaria M, Feo F, Pascale RM, Prieto J, Berasain C, Avila MA. Regulation of amphiregulin gene expression by beta-catenin signaling in human hepatocellular carcinoma cells: a novel crosstalk between FGF19 and the EGFR system. PLoS One. 2012; 7:e52711. https://doi.org/10.1371/journal.pone.0052711. [PubMed]
5. Xu C, Liu C, Huang W, Tu S, Wan F. Effect of Mst1 overexpression on the growth of human hepatocellular carcinoma HepG2 cells and the sensitivity to cisplatin *in vitro*. Acta Biochim Biophys Sin (Shanghai). 2013; 45:268–79. https://doi.org/10.1093/abbs/gmt006. [PubMed]
